# Disorganized thalamic subregional functional connectivity in bipolar I disorder

**DOI:** 10.1002/mco2.771

**Published:** 2024-10-31

**Authors:** Xipeng Long, Xiuli Wang, Yuan Cao, Di Kong, Baolin Wu, Hongsheng Xie, Ziru Zhao, Neil Roberts, Qiyong Gong, Zhiyun Jia

**Affiliations:** ^1^ Department of Nuclear Medicine West China Hospital of Sichuan University Chengdu China; ^2^ Research Unit of Psychoradiology Chinese Academy of Medical Sciences Chengdu China; ^3^ Department of Psychiatry the Fourth People's Hospital of Chengdu Chengdu China; ^4^ Department of Radiology and Huaxi MR Research Center (HMRRC) Functional and Molecular Imaging Key Laboratory of Sichuan Province West China Hospital of Sichuan University Chengdu China; ^5^ The Queens Medical Research Institute (QMRI) School of Clinical Sciences University of Edinburgh Edinburgh UK; ^6^ Department of Radiology West China Xiamen Hospital of Sichuan University Xiamen China

**Keywords:** bipolar disorder, functional connectivity, subcortical, subregion, thalamus

## Abstract

Thalamus plays a pivotal role in the pathophysiology of neuropsychiatric conditions due to its strategic position and intricate connectivity with the cerebral cortex, limbic system, and other subcortical structures. In the present study, the potential involvement of the thalamus and subregions of the thalamus are explored in bipolar disorder (BD). In particular, functional and structural magnetic resonance imaging was performed on 73 adult patients with BD‐I and 78 healthy controls (HCs). Seed‐based thalamus and thalamic subregional functional connectivity (FC) were compared between the BD‐I patients and HCs. Compared to HCs, patients with BD‐I showed higher FC between the left thalamus and right lingual gyrus and altered FC between the dorsal thalamus and the default mode network and prefrontal regions, which may be correlated with mania symptomatology. In patients with BD‐I, the anterior subregions of the thalamus had higher FC than the posterior subregions. No significant difference in gray matter volume or local functional activity was found in the thalamus and thalamic subregions between BD‐I and HC. These findings provide evidence of disorganized thalamocortical FC in BD‐I, suggesting that the thalamus and its subregions may play important and specific roles in the neural circuitry of BD.

## Graphical abstract

**FIGURE 1** Flowchart of inclusion and exclusion criteria, and resting‐state functional connectivity (FC) analysis of subcortical regions and thalamic subregions. A total of 151 participants were enrolled. All participants were scanned with high‐resolution MRI. After data collection, preprocessing and seed‐based FC analysis were then performed. MRI, magnetic resonance imaging.

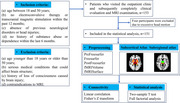

## 1 INTRODUCTION

Bipolar disorder (BD) is a debilitating neuropsychiatric disorder characterized by multiple manifestations of emotional episodes, including typical depressive and manic/hypomanic episodes, as well as mixed states. These episodes profoundly affect the lives of patients and their families, leading to significant impairment.^1^ Individuals with BD also experience psychotic symptoms, which is characteristic of the schizophrenia (SCZ)‐bipolar spectrum. Therefore, individuals with BD exhibit diverse symptoms including mood fluctuations, sensorimotor dysfunction, and cognitive decline as observed in mood disorders and psychosis. These symptoms arise from dysfunctions in multiple brain systems.^2^, ^3^ Among these systems, the thalamus plays a critical role as a central hub in regulating various aspects of emotion, sensory perception, motor function, and cognition, given its extensive connections with cortical, subcortical, and cerebellar regions. Thus, the thalamus has been implicated in the pathology of numerous psychiatric disorders. However, compared to other psychiatric disorders such as major depressive disorder (MDD) and SCZ, the role of the thalamus in BD remains relatively less understood and is a particular focus of the present study.

It is important to note that abnormalities in the thalamus play a significant role in the occurrence and progression of BD. Individuals with BD have reduced thalamic volume compared to healthy people, and larger thalamic volume in individuals with BD taking lithium.^4^, ^5^ This suggests that the thalamus may be a potential target for medication. Previous studies have consistently shown a distinct pattern of thalamic functional connectivity (FC) in psychosis, characterized by increased connectivity with sensory areas such as the motor cortex, temporal cortex, and occipital area, and lower connectivity with prefrontal and cerebellar regions.^6^ These connectivity patterns correspond to abnormalities in sensory experience and attribution, motor coordination, and neuropsychological function.^7^ BD is an important part of the psychosis spectrum, however, studies investigating the role of the thalamus in BD are limited and the results remain inconsistent.

The thalamus is not a homogeneous structure but rather comprises heterogeneous nuclei with overlapping or different projections to cortical fields. Few studies have investigated how specific thalamic subnuclei or subregions contribute to alterations in BD. Recent research has revealed smaller volumes in the mediodorsal, pulvinar, and lateral and medial geniculate thalamic nuclei in BD, with a history of psychosis associated with smaller volumes of the mediodorsal nucleus.^8^ In terms of functional studies, one study did not find any thalamocerebellar dysconnectivity in BD, which differs from findings in SCZ.^9^ Another study found decreased FC between the dorsomedial thalamus and the pregenual anterior cingulate cortex in BD, which was consistent across mood episodes but exhibited a more severe pattern compared to MDD.^10^ These findings suggest that individuals with BD, as a psychiatric disorder encompassing both mood and psychotic features, exhibit unique thalamo‐cortico FC. Therefore, investigating alterations in thalamic FC and structure is necessary for a deeper understanding of the pathophysiology of BD.

The main objective of the present study was to apply a seed‐based method using a new thalamic subregional atlas to investigate the potential role of the thalamus and subregions of the thalamus in each cerebral hemisphere in alterations in brain FC in patients with BD‐I compared to healthy controls (HCs). A secondary objective was to investigate whether alterations in brain connectivity were associated with alterations in brain anatomy and disease severity.

## 2 RESULTS

### 2.1 Demographic characteristics

**FIGURE 1:**
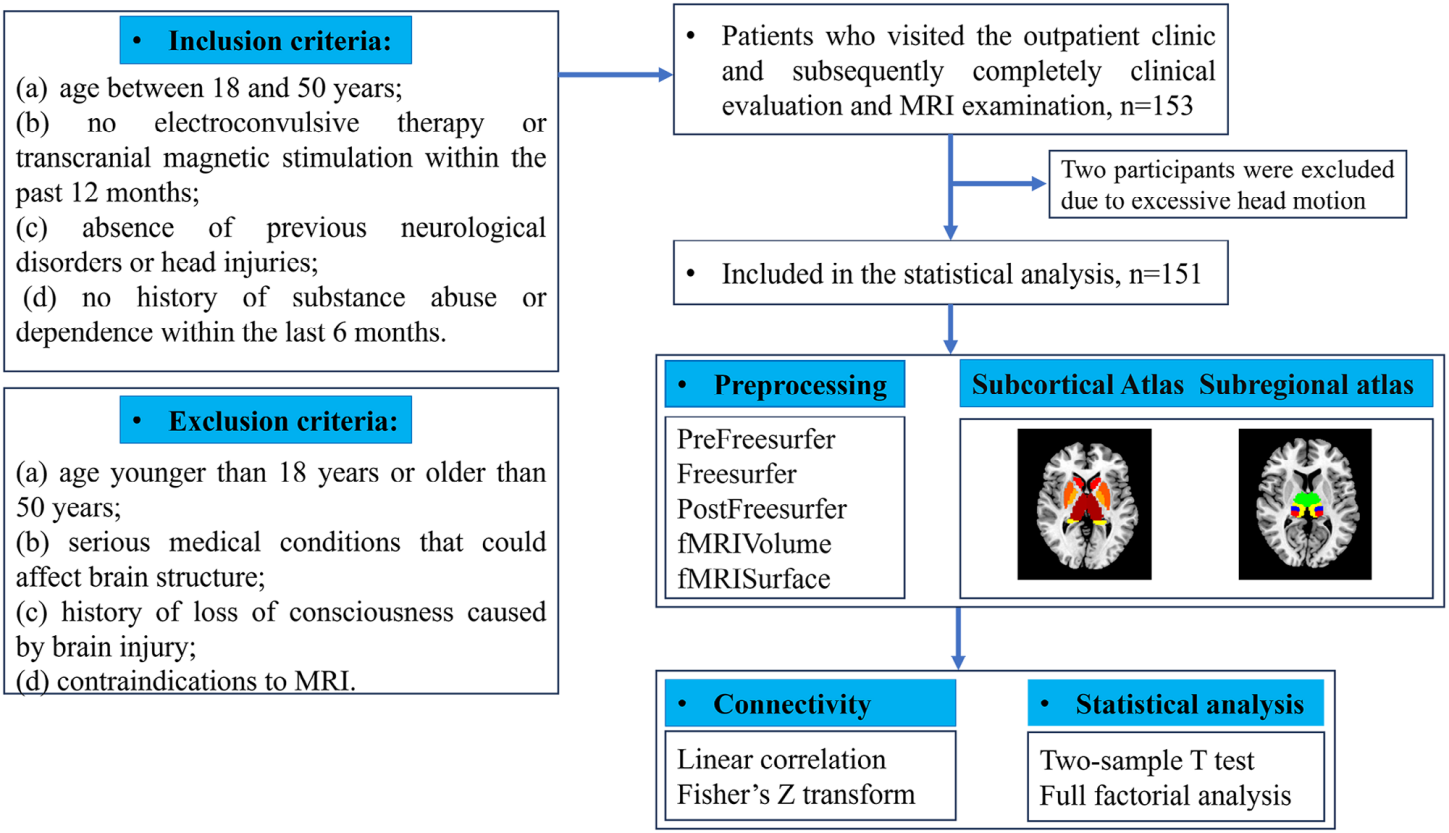
Flowchart of inclusion and exclusion criteria, and resting‐state functional connectivity analysis of subcortical regions and thalamic subregions. A total of 151 participants were enrolled. All participants were scanned with high‐resolution MRI. After data collection, preprocessing and seed‐based functional connectivity analysis were then performed. MRI, magnetic resonance imaging.

One patient with BD‐I and one HC were excluded due to a mean framewise displacement (FD) value greater than 0.2 mm, and thus results are presented for 73 patients with BD‐I and 78 HCs (Figure 1). Of the patients with BD‐I, 39 were currently experiencing a depressive episode, 14 euthymia, 15 manic, and five mixed episodes. The demographic and clinical characteristics of the three groups are listed in Table 1. There were no significant differences in age or sex between BD‐I patients and HCs. BD‐I patients had significantly fewer education years than HCs, and the level of education was included as a potential influencing factor in the covariate analysis.

**TABLE 1:** Demographics and clinical characteristics of patients with bipolar disorder (BD)‐I and healthy controls (HCs).

	BD‐I	HCs	BD‐I versus HCs	
	*n* = 73	*n* = 78	T‐Values	*p*‐values
*Demographic characteristics*				
Age, years	25.51 (6.08)	27.54 (7.70)	−1.792	0.075
Male (%)	19 (26.03%)	24 (29.00%)	0.416	0.642
Education, years	14.41 (2.71)	17.60 (2.33)	−7.79	<0.001
*Clinical characteristics*				
Illness duration, years	6.92 (4.23)	–	–	–
Onset age, years	18.53 (5.77)	–	–	–
HAMD‐17	11.99 (7.39)	0.90 (1.65)	12.92	<0.001
HAMA	14.05 (9.26)	0.77 (1.55)	12.49	<0.001
YMRS	6.84 (7.82)	0	7.72	<0.001
PANSS	40.04 (9.27)	30.08 (0.27)	9.49	<0.001
*Current medication use, n (%)*	66 (90.41%)	0	–	–
Mood stabilizers	56 (76.71%)	–	–	–
Antidepressants	33 (45.21%)	–	–	–
Antipsychotics	46 (63.01%)	–	–	–
Benzodiazepines	20 (27.40%)	–	–	–

Abbreviations: BD‐I, bipolar disorder type I; HAMA, Hamilton Anxiety Scale; HAMD, Hamilton Depression Scale; HCs, healthy controls; YMRS, Young, Manic Rating Scale; PANSS‐N, Positive and Negative Syndrome Scale‐Negative; PANSS‐G, Positive and Negative Syndrome Scale‐General; PANSS‐P, Positive and Negative Syndrome Scale‐Positive.

### 2.2 Between‐group differences in thalamic seed‐based FC

**FIGURE 2:**
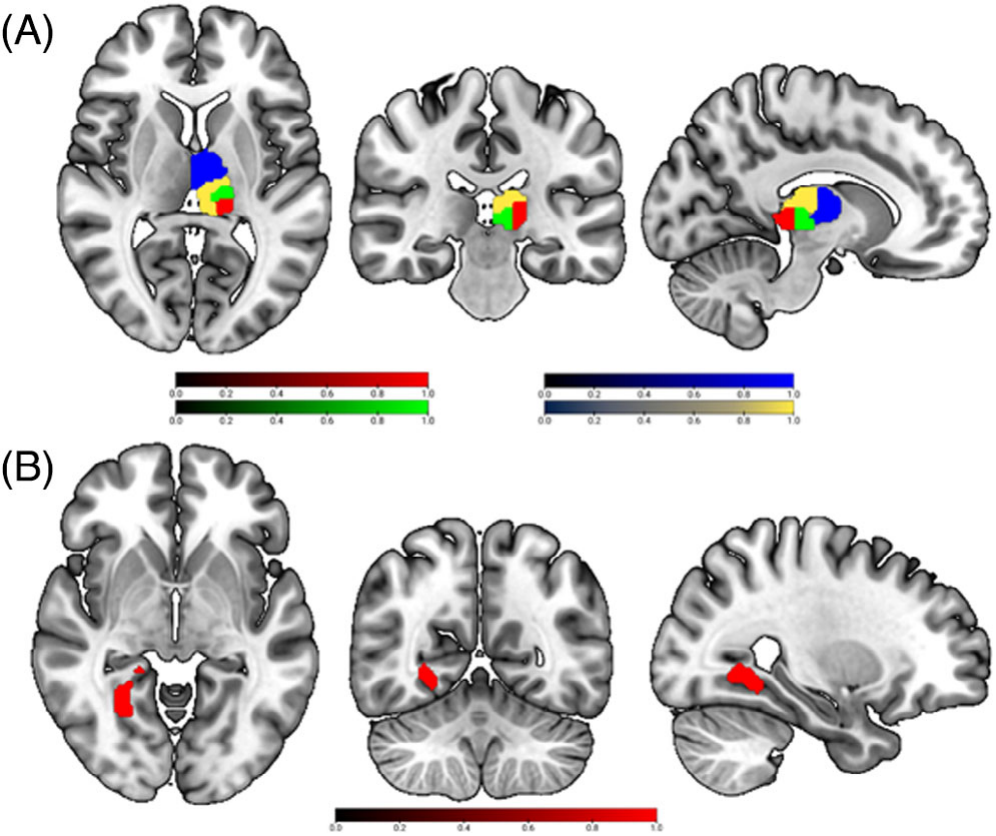
Atlas of thalamic subregions and results of group comparison of subcortical rsFC. (A) Functional gradient parcellation of the thalamus in the current study. (B) BD‐I patients showed higher rsFC between the left thalamus and right lingual gyrus compared to HCs. BD‐I, bipolar disorder type I; HC, healthy controls; rsFC, resting‐state functional connectivity. This Figure was created using MRIcron. (https://www.nitrc.org/projects/mricron/).

Compared to HCs, BD‐I patients showed higher FC between the left thalamus and right lingual gyrus (Table 2 and Figure 2B). These results remained significant after including lithium load, medication load, and mean FD as covariates.

**TABLE 2:** Brain regions with significant differences in seed‐based functional connectivity of subcortical regions and thalamic subregions between bipolar disorder (BD)‐I group and healthy controls (HCs).

Seed regions	Area	Cluster	MNI coordinate (x, y, z)	T/F value	Group Difference
**BD‐I**					
**Subcortex**					
L Thalamus	R Lingual Gyrus	235	26, −58, −4	−4.159	BD‐I > HCs
**Subregion × group**					
	L anterior cingulate cortex	112	0, 46, 8	9.56582	
	L superior frontal gyrus	124	−26, 64, 2	8.4832	
	L middle frontal gyrus	124	−28, 42, 24	7.896	
	L angular gyrus	297	−42, −68, 42	9.1085	
	L middle cingulate	144	−2, −24, 36	9.4799	
	R precuneus	122	6, −74, 42	11.3552	

Abbreviations: BD‐I, bipolar disorder type I; HCs, healthy controls; L, left; MNI, Montreal Neurological Institute; R, right; rsFC, resting‐state functional connectivity.

### 2.3 Between‐group and within‐group differences in thalamic subregional seed‐based FC

Inspection of the results from the full factorial analysis of the FC of the four seeds in subregions of the thalamus revealed significant group × thalamic subregions interaction effects in the left cingulate cortex, left superior frontal gyrus, left middle frontal gyrus, left angular gyrus, left middle cingulate gyrus and right precuneus in BD‐I patients (Table 2 and Figure 2B). No significant interaction effects were found between group × direction, direction × subregions, or among group × direction × subregions. All the results remained significant after including lithium dose, medication load, and mean FD as covariates.

**FIGURE 3:**
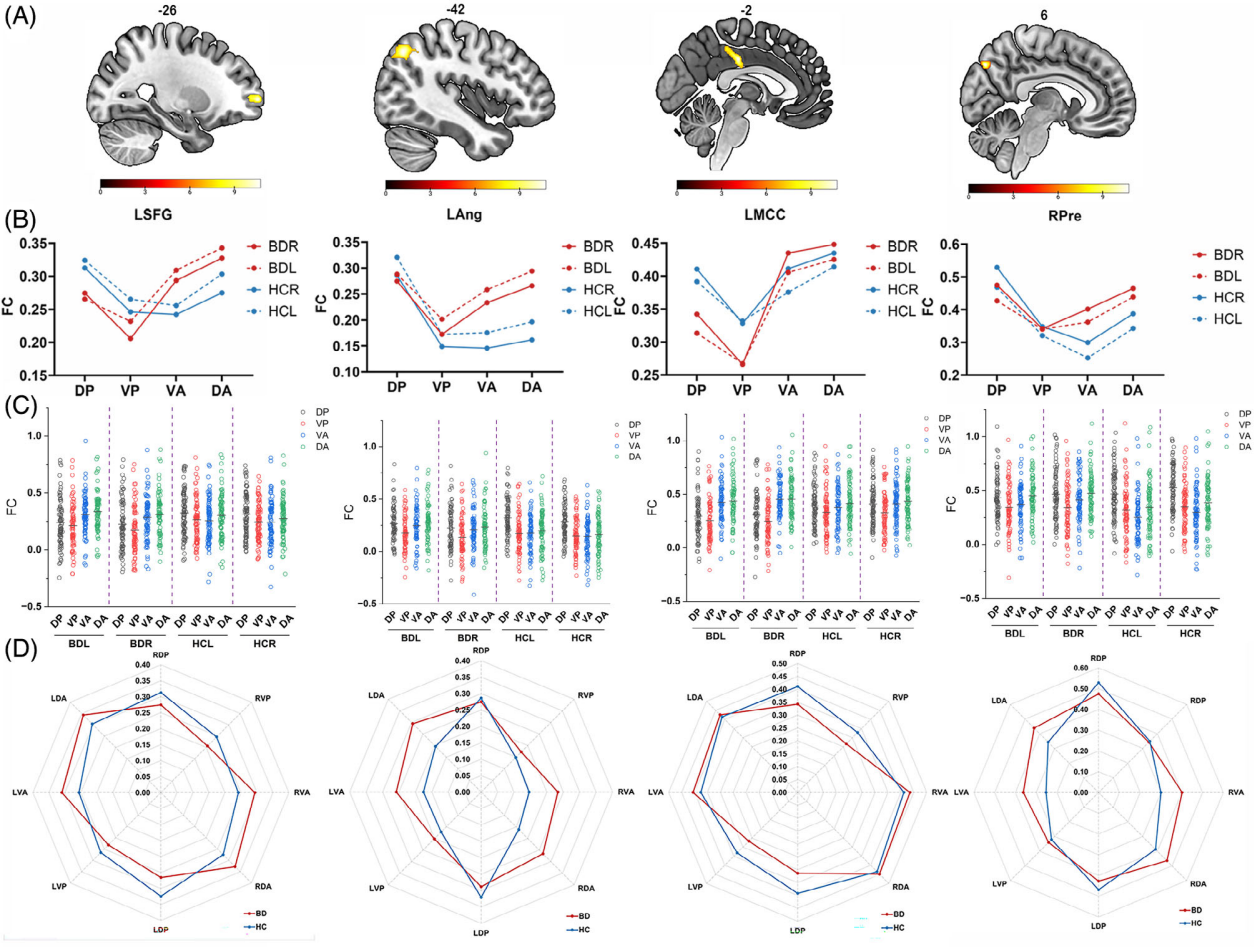
Thalamic subregional rsFC in BD‐I patients (*n* = 73) and HCs (*n* = 78). (A) Full factorial analysis revealed significant interactions between group (BD‐I vs. HC) and thalamic subregions (DP vs. VP vs. VA vs. DA) in the LSFG, Lang, LMCC, and RPre. (B) Significant interactions between group and thalamic subregions. This was created using Graphpad Prism 9.5 (https://www.graphpad.com/scientific‐software/prism/www.graphpad.com/scientific‐software/prism/). (C) Simple effect tests comparing rsFC between‐group and within‐group. This was created using Origin (https://www.originlab.com/). (D) Polar plots illustrating thalamic subregional rsFC showing between‐group and within‐group differences in BD‐I patients and HCs This was created using easy charts (https://github.com/EasyChart/Excel‐Chart‐Plugin‐EasyCharts). BD‐I, bipolar disorder type I; HCs, healthy controls; DP, dorsal posterior; VP, ventral posterior; VA, ventral anterior; DA, dorsal anterior; LSFG, left superior frontal gyrus; Lang, left angular gyrus; LMCC, left middle cingulate cortex; RPre, right precuneus; BDR, bipolar disorder‐right; BDL, bipolar disorder‐left; HCR, healthy controls‐right; HCL, healthy controls‐left; RDP, right dorsal posterior; LDP, left dorsal posterior; RDA, right dorsal anterior; LDA, left dorsal anterior; RVP, right ventral posterior; LVP, left ventral posterior; RVA, right ventral anterior; LVA, left ventral anterior.

The results of simple post‐hoc tests, incorporating correction for multiple comparisons to obtain details about which thalamus subregions contribute to the between‐group effects reported above are presented in Table 3. Patients with BD‐I showed higher FC between the left superior frontal gyrus and dorsal posterior (DP) (*F* = 7.25, *p* = 0.008, *η* ^2 ^= 0.053); between left angular gyrus and dorsal anterior (DA) (*F* = 6.65, *p* = 0.011, *η* ^2 ^= 0.042); between left middle cingulate gyrus and DP (*F* = 9.42, *p* = 0.003, *η* ^2 ^= 0.059), and between the right precuneus and ventral anterior (VA) (*F* = 11.33, *p* = 0.001, *η* ^2 ^= 0.071) and DA (*F* = 7.96, *p* = 0.005, *η* ^2 ^= 0.050) compared to HCs (Figure 3B,C).

**TABLE 3:** Simple effect tests comparing resting‐state functional connectivity (rsFC) between bipolar disorder (BD)‐I patients and healthy controls (HCs) in each thalamic subregion.

Subregions	BD‐I			
	Regions	BD‐I	HCs	*p*‐value
DP	LACC	0.274 (0.027)	0.365 (0.026)	0.017
VP		0.264 (0.028)	0.322 (0.027)	0.136
VA		0.463 (0.025)	0.392 (0.024)	0.043
DA		0.419 (0.026)	0.372 (0.025)	0.194
DP	LSFG	0.231 (0.023)	0.319 (0.022)	**0.008** ^*^
VP		0.194 (0.022)	0.256 (0.021)	0.042
VA		0.295 (0.022)	0.249 (0.021)	0.125
DA		0.323 (0.021)	0.289 (0.021)	0.257
DP	LMFG	0.232 (0.025)	0.310 (0.024)	0.028
VP		0.202 (0.024)	0.246 (0.023)	0.186
VA		0.273 (0.023)	0.213 (0.022)	0.063
DA		0.297 (0.024)	0.234 (0.023)	0.064
DP	LAng	0.245 (0.020)	0.304 (0.019)	0.039
VP		0.156 (0.020)	0.161 (0.019)	0.868
VA		0.228 (0.021)	0.161 (0.020)	0.021
DA		0.256 (0.022)	0.179 (0.021)	**0.011** ^*^
DP	LMCC	0.301 (0.023)	0.401 (0.023)	**0.003** ^*^
VP		0.252 (0.023)	0.330 (0.022)	0.014
VA		0.439 (0.022)	0.393 (0.021)	0.143
DA		0.445 (0.023)	0.425 (0.022)	0.526
DP	RPre	0.450 (0.025)	0.499 (0.025)	0.165
VP		0.342 (0.025)	0.334 (0.024)	0.836
VA		0.392 (0.025)	0.276 (0.024)	**0.001** ^*^
DA		0.463 (0.025)	0.365 (0.024)	**0.005** ^*^

Abbreviations: BD‐I, bipolar disorder type I; DA, dorsal anterior; DP, dorsal posterior; HCs, healthy controls; L, left; LACC, left anterior cingulate cortex; LAng, left angular gyrus; LMCC, left middle cingulate cortex; LSFG, left superior frontal gyrus; MNI, Montreal Neurological Institute; R, right; RPre, right precuneus; rsFC, resting‐state functional connectivity; RSFG, right superior frontal gyrus; VA, ventral anterior; VP, ventral posterior.

* indicates significant results that survived multiple comparison corrections (Bonferroni).

Exploratory analysis revealed a significant positive partial correlation between FC between left DP and left superior frontal gyrus and YMRS scores (*r* = 0.247, *p* = 0.039); between left MCC and right DP and YMRS scores (*r* = 0.296, *p* = 0.013). A significant negative partial correlation was found between FC between the right precuneus and right DA and PANSS‐N scores (*r* = −0.275, *p* = 0.021). However, these correlations did not survive multiple comparison corrections. We also found that manic patients showed higher FC between the left superior frontal gyrus and left (*F* = 6.944, *p* < 0.001, *η* ^2^ = 0.240) and right DP (*F* = 6.460, *p* = 0.001, *η* ^2 ^= 0.227); between left angular gyrus and right DA compared to depressive and euthymic patients (*F* = 6.499 *p* = 0.001, *η* ^2 ^= 0.228). Manic patients also showed higher FC between left middle cingulate gyrus and left (*F* = 8.455, *p* < 0.001, *η* ^2 ^= 0.278) and right DP (*F* = 6.998, *p* < 0.001, *η* ^2 ^= 0.241); between right precuneus and left (*F* = 5.182, *p* = 0.003, *η* ^2 ^= 0.191) and right DA (*F* = 4.535, *p* = 0.006, *η* ^2 ^= 0.171) compared to depressive and euthymic patients (Table S1). Another interesting finding was that patients who receive three kinds of medication showed lower FC between the left angular gyrus and right DA compared to patients receiving only one kind of medication (patients receiving one kind of medication = 0.391 ± 0.065, patients receiving three kinds of medication = 0.161 ± 0.034; *F* = 4.680, *p* = 0.001, *η* ^2 ^= 0.263); patients receiving only one kind of medication showed higher FC between the right precuneus and left VA compared to patients receiving two and three kinds of medication (patients receiving one kind of medication = 0.619 ± 0.072, patients receiving two kinds of medication = 0.285 ± 0.039, patients receiving three kind of medication = 0.335 ± 0.037; *F* = 4.680, *p* = 0.001, *η* ^2 ^= 0.259) (Table S2).

A similar analysis as the above was performed to analyze the within‐group effects. In BD‐I patients, this revealed (i) stronger FC of DA (*F* = 28.513, *p* < 0.001) with left anterior cingulate cortex compared to DA, ventral posterior (VP), and DP, (ii) stronger FC of DA with left superior frontal gyrus (*F* = 13.628, *p* < 0.001) and middle frontal gyrus (*F* = 5.952, *p* < 0.001) compared to VP, (iii) stronger FC of DP (*F* = 9.369, *p* < 0.001) with left angular compared to VP, (iv) stronger FC of VA (*F* = 35.774, *p* < 0.001) with left middle cingulate gyrus compared to VP and DP, and (v) weaker FC of VP with right precuneus (*F* = 9.489, *p* < 0.001) compared to DP and DA (Seen in Figure 3D, Table S3).

In HCs, within‐group analysis revealed (i) stronger FC of VA with left anterior cingulate cortex (*F* = 2.619, *p* < 0.001) compared to VP, (ii) stronger FC of DP with left superior frontal gyrus (*F* = 4.374, *p* < 0.001) compared to VP and VA, (iii) weaker FC of DP with right superior frontal gyrus (*F* = 6.306, *p* < 0.001) compared to VA and DA, (iii) stronger FC of DP with left angular gyrus (*F* = 23.335, *p* < 0.001) compared to VP, VA, and DA, and (iv) weaker VP FC with the left middle cingulate gyrus (*F* = 6.536, *p* < 0.001) compared to DP, VA, and DA, (v) stronger FC between right precuneus (*F* = 28.907, *p* < 0.001) and DP compared to VP, VA, and DA (Figure 3D, Table S3).

### 2.4 Between‐group differences in gray matter volume and local activity

Surface‐based analysis revealed smaller right pulvinar gray matter (GM) volume in BD‐I patients compared to HCs (*F* = 5.838, *p* = 0.017, *η* ^2 ^= 0.039). However, this did not survive multiple comparison corrections. In terms of the amplitude of low‐frequency fluctuations (ALFF) and fractional ALFF (fALFF) values, BD patients showed reduced ALFF values in the left VP compared to HC (*F* = 6.148, *p* = 0.014). No significant difference in fALFF values for BD compared with HC.

## 3 DISCUSSION

Comparison of subcortical FC in patients with BD‐I and HCs has shown that (1) FC is significantly higher between the left thalamus and right lingual gyrus in patients with BD‐I compared to HCs, and when subregions of the thalamus are considered (2) with regard to between‐group effects there is significantly higher FC between anterior thalamic subregions bilaterally and left superior frontal gyrus, angular gyrus, middle cingulate gyrus, and right precuneus in BD‐I patients compared to HC and (3) with regard to within‐group effects, there is stronger FC in anterior subregions of the thalamus than in other subregions in BD‐I patients and in dorsal posterior subregions of the thalamus than in other subregions in HCs.

The observation of higher FC between the left thalamus and right lingual gyrus in patients with BD‐I is consistent with previous research on BD. Previous studies have reported higher FC between the thalamus and sensory areas, such as the motor cortex, temporal cortex, and occipital cortex, in individuals with BD.^11^ Moreover, the thalamus has been found to exhibit higher activation during cognitive and emotional tasks, independent of mood episodes.^12^, ^13^ The lingual gyrus is located in the medial part of the occipitotemporal gyrus and is an important region of the visual cognitive network involved in processing faces and emotion recognition.^14^ The lingual gyrus plays a crucial role in the specific pathology of BD.^15^ and the altered function of the lingual gyrus in BD patients has been found to be related to the deficits of visual sensory processing and episodic memory.^16^, ^17^ A possible interpretation of the finding of higher connectivity between the thalamus and lingual gyrus is that this may be the result of neuroplastic changes within the brain. These findings suggest that there may be aberrant neural circuits involved in relaying and integrating sensory information in individuals with BD, contributing to the observed alterations in FC between the thalamus and lingual gyrus.^11^ Further research is needed to better understand the underlying mechanisms and implications of these connectivity changes in the context of BD.

Analysis of thalamic subregional FC revealed lower FC between DP (medial pulvinar nucleus, PuM) and left superior frontal gyrus and left middle cingulate gyrus; higher FC between DA (central lateral [CL] and LP nuclei) and left angular gyrus and right precuneus; and higher FC between the VA (MD nucleus) and right precuneus in BD‐I patients. Lower FC between the thalamus and prefrontal regions aligns with the literature on BD and psychosis. Previous studies also suggested that manic BD patients had thalamo‐frontal hyperconnectivity and euthymic BD patients had thalamo‐frontal hypoconnectivity, which is consistent with our findings.^18^ Superior frontal gyrus and middle cingulate cortex contribute to emotion regulation and motor control in BD, respectively.^19^ However, the role of the medial pulvinar nucleus in BD remains largely understudied. Considering the reciprocal connectivity of the pulvinar with the posterior parietal, inferior temporal, cingulate, and prefrontal cortical regions, it can be inferred that the medial pulvinar acts as a multimodal association hub, playing a role in the regulation and focus of attentional orientation.^20^ The lower FC between the superior frontal gyrus, middle cingulate cortex, and the medial pulvinar nucleus of the thalamus may indicate an impairment in bottom‐up attention and emotion regulation. Furthermore, our findings support that the feedback loops involving the prefrontal and thalamus might serve as a significant pathophysiological indicator in BD.

The precuneus showed higher FC to DA and VA nucleus of the thalamus. The anterior part of the precuneus belongs to the anterior subsystem of default mode network (DMN), which is thought to facilitate mental theory and self‐referential thinking; the posterior part of the precuneus belongs to the posterior subsystem of DMN, which is associated with episodic memory and visual‐spatial imagery.^21^ The higher connectivity between the right precuneus and VA and DA may indicate its segregation function role in BD. We also found that manic patients showed higher thalamic subregional FC compared to depressive and euthymic patients, especially between dorsal thalamic nuclei and DMN regions, suggesting manic patients may have an abnormal coupling between dorsal thalamus and DMN oscillations. Previous studies also revealed increased FC within DMN regions and between DMN and other large‐scale brain networks.^22^ This might allow an over‐influence of goal‐directed inputs and a predominance of DMN onto ongoing intrinsic activity, which may symptomatically result in intrinsic impulsive behavior and flight of thought.^23^

The angular gyrus is a cross‐modal hub, allowing both internal and external sources to gain access to conceptual understandings of events or objects within their special spatial and temporal frameworks.^24^ Alterations in this region have been observed in different modalities in BD, including thinner cortical thickness^25^ and higher participation coefficients.^26^ The CL nuclei of the thalamus have been proposed to play a crucial role in supporting consciousness.,^27^ and the LP nuclei of the thalamus are reciprocally connected with sensorimotor areas.^28^ The higher FC between the DA and the angular gyrus in BD‐I may suggest an overactivity in the top‐down regulation of internal and external inputs in BD patients, which may be more prevalent in manic states.

The within‐group analysis of subregions of the thalamus revealed distinct patterns of FC in BD‐I and HC. In BD‐I, there was stronger FC observed between anterior thalamic subregions (MDpc, VApc, and CL nuclei) and prefrontal cortex as well as regions within the DMN. These anterior subregions of the thalamus have connections with the frontal cortex, cingulate cortex, motor and premotor cortex, and somatosensory cortex. They are primarily involved in visual and sensorimotor functions.^29^, ^30^ This higher connectivity between anterior thalamic subregions and prefrontal/DMN regions in BD suggests altered information processing and integration between these regions involved in cognitive and emotional functions. On the other hand, in HC participants, there was a trend for stronger FC observed in dorsal subregions of the thalamus, particularly the DP nucleus (part of the pulvinar nucleus), compared to other subregions. The pulvinar is the largest thalamic nucleus and is involved in various functions, including visual, auditory, and somatic information processing, attention, emotional association, and language.^31^ The medial part of the pulvinar is thought to be involved in emotional processes and saliency detection.^32^ This differential connectivity pattern in HC, with stronger connectivity in dorsal subregions, may reflect the integration of visual, sensorimotor, and emotional processing in a more organized manner. The observed differences in FC patterns between BD‐I and HC suggest disrupted thalamic subregional connectivity, particularly along the anterior‐posterior axes, in BD‐I patients. These findings provide further evidence of altered thalamic connectivity patterns in BD‐I and highlight the importance of considering specific subregions of the thalamus when studying FC in the disorder.

In the current study, patients with BD‐I have lower education years compared to healthy controls. The first episode of BD often occurs in adolescence in our included participants, and individuals with server symptoms might suspend or drop out of school. This might be an important reason for the lower education year in individuals with BD‐I. The education and learning process also strongly depends on age, and studies have revealed that pruning and myelination occur in the central nervous system during learning and education.^33^ In terms of the thalamus and its subregions, they serve as a central hub for functional brain networks, playing a crucial role in the mediation of many cognitive and executive functions. One study found enhanced FC between the thalamus and the frontoparietal control network in chess players with a high level of intensive cognitive training.^34^ This evidence suggests that the function of the thalamus can be closely related to cognition, which is also the core of the learning process and education. Our study added the education year as a covariate to minimize its impact, however, investigating the impact of education level still needs future work.

Interestingly, the observed changes in FC of the thalamus and its subregions in BD‐I were not found to be related to differences in GM volume. The finding aligns with a review.^35^ that reports alterations in thalamus function without corresponding structural alterations in most studies. Although it has been reported in recent studies that the volume of the thalamus is increased.,^36^ and decreased,^4^ in patients with BD compared to HCs, few studies have investigated the GM volume of thalamic subnuclei. Alteration in the function of the thalamus in BD may not be accompanied by structural changes or perhaps it is the case that functional alterations precede structural changes.

In an exploratory analysis, we found that patients receiving three kinds of medication showed lower FC between thalamic subregions and angular gyrus and precuneus. This finding suggests that a combination of medications may have a protective effect on symptom onset, especially in mania. However, this result needs careful interpretation since it is an exploratory analysis and the effect size was small. Although no significant medication effect was observed after adding them as covariates, medication‐naïve patients should ideally be recruited in further studies. Considering the complexity of medication regimens among outpatient patients, we ensured that all the patients were on regular medication one week before and after the magnetic resonance imaging (MRI) scan, therefore, we only evaluated medication effects according to the medication category. In addition, almost all patients received a combination of medications including mood stabilizers, antipsychotics, and antidepressants, therefore it is hard to purely carry out a subgroup analysis focusing on only one medication. In the future, there will be a need for detailed differentiation of medication timing and the effects of different drugs on patients.

The present study has several limitations that should be acknowledged. First, the study is cross‐sectional and a longitudinal design would be required to study causal effects. Secondly, the sample size used in this study is modest, therefore, the conclusions obtained from this study must be replicated in relatively larger samples in order to improve reliability and reproducibility, which is critical in neuroscience.^37^ Thirdly, the atlas that was used to define the subregions of the thalamus in this study can fully unveil the topographic organization of the thalamus and its subregions. However, other probabilistic atlases of thalamic nuclei may provide thalamic FC profiles from a different perspective.

## 4 METHODS

### 4.1 Participants

The authors assert that all procedures contributing to this work comply with the ethical standards of the relevant national and institutional committees on human experimentation and with the Helsinki Declaration of 1975, as revised in 2008. All procedures involving human patients were approved by the Research Ethics Committees of West China Hospital of Sichuan University (study ethical approval number: 2020[331]). A total of 153 individuals comprising 74 BD patients and 79 HCs were recruited through the Department of Psychiatry of the Fourth People's Hospital of Chengdu, China between 2019 and 2022, and demographic and clinical information is presented in Table 1. All participants provided fully informed written consent of their willingness to participate.

Diagnosis of BD was established based on the Structured Clinical Interview for DSM‐V diagnostic criteria (SCID) by two professional psychiatrists (Xipeng Long and Di Kong). Inclusion criteria for patients were as follows: (a) age between 18 and 50 years, (b) no electroconvulsive therapy or transcranial magnetic stimulation within the past 12 months, (c) absence of previous neurological disorders or head injuries, and (d) no history of substance abuse or dependence within the last 6 months. HCs were recruited through advertising and met the following criteria: (a) no current or past diagnosis of any psychiatric disorders and (b) no family history of psychiatric disorders. Exclusion criteria for all participants were: (a) age younger than 18 years or older than 50 years, (b) serious medical conditions that could affect brain structure, (c) history of loss of consciousness caused by brain injury, and (d) contraindications to MRI. The severity of depression, anxiety, (hypo)mania, and psychotic symptoms in all participants was assessed using standardized rating scales, including the 17‐item Hamilton Depression Scale (HAMD), 14‐item Hamilton Anxiety Scale (HAMA), Young Mania Rating Scale (YMRS), and Positive and Negative Syndrome Scale (PANSS), respectively.

### 4.2 MRI data acquisition and preprocessing

MRI was performed on a 3.0 T system (Trio, Siemens Healthineers) equipped with a 32‐channel head coil. Detailed acquisition parameters are provided in the Supporting Information material.

Image analysis was carried out on a Linux Ubuntu (18) computer with the following software installed: HCPpipelines (v4.2.1), MATLAB MCR (v2017b), and R (v4) software. The data preprocessing followed the minimal pipeline recommended by the Human Connectome Project for HCP‐style data. Further details regarding the preprocessing methods can be found in the Supporting Information material. A flowchart illustrating the study procedures is provided in Figure 1

### 4.3 Seed‐based thalamus FC analysis

First, the rs‐fMRI images that met the criteria of <1.5 mm of spatial movement and <1.5 degrees of rotation in any direction and a mean FD value <0.2 mm were included in subsequent analyses (one patient and one HC were excluded). Then, seed‐to‐voxel FC analyses were performed in DPABI using the Harvard‐Oxford Atlas‐based subcortical seed regions. The average blood oxygenation level‐dependent signal in each region of interest (ROI) was extracted and correlated with the time course of each voxel throughout the brain. The resulting correlation coefficients were then transformed into normally distributed scores using Fisher's r‐to‐z transformation. Voxel‐based *z*‐value maps of the thalamus regions were compared between patient groups and HCs, with age, sex, and education years as covariates, by using a random‐effect two‐sample *t*‐test in DPABI. To control for false positives, the significance threshold was set to *p* < 0.001 at the voxel level, and correction for multiple comparisons was performed by using cluster‐level family‐wise error (FWE) thresholding (*p* < 0.05).

### 4.4 Thalamic subregion seed‐based FC analysis

Four thalamic subregions were considered in each cerebral hemisphere, namely DA, VA, DP, and VP subregions. The relevant ROIs were created using subcortical cartography maps defined by gradientography using ultrahigh field MRI.^38^ This relevant atlas is available online (https://github.com/yetianmed/subcortex). The four subregions that have been chosen for analysis in the present study may be mapped to one or a cluster of histologically delineated nuclei. The DP of the thalamus may be considered to map to the PuM, the VP to the central median and VP lateral nucleus (VPLp), the VA to the mediodorsal nucleus parvocellular part (MDPc) and VA nucleus parvocellular part (VAPc), and the DA to the CL nucleus and lateral posterior nucleus (LP).^38^

By using SPM 12, the FC maps for each of the 14 subcortical seed regions used in the individual‐level analyses were submitted to a second‐level group analysis that treated participants as a random variable in a 2 × 2 × 4 full factorial analysis of variance, with group (BD‐I vs. HCs) as a between‐group factor and subregions (DP vs. VP vs. VA vs. DA) and hemispheres (left vs. right) as within group factors. To control for potential false positives, the significance threshold was set to *p* < 0.001 at the voxel level, and correction was made for multiple comparisons by using cluster‐level FWE thresholding (*p* < 0.05), which is the recommended method based on random field theory.^39^, ^40^ For brain regions showing significant interaction effects, simple post‐hoc tests were employed with Bonferroni correction for multiple comparisons to obtain details about which thalamus subregions contribute to the between‐group and within‐group effects.

### 4.5 Surface‐based morphometry analysis and local activity analysis

To investigate potential anatomical changes associated with the observed functional abnormalities, the GM volume of the thalamus and its subregions was quantified using surface‐based morphometry in Freesurfer. The preprocessing of T1 images is detailed in the Supporting Information material. Comparison of GM volume in each thalamic subregion in patients and HCs was conducted by using multivariate analysis of covariance with age, sex, and education years as covariates in the general linear model.

To explore whether the FC aberrations were accompanied by or associated with alterations in local activity, the ALFF and fALFF values of the thalamus and its subregions were calculated in DPABI. The differences in local activity of thalamic subnuclei were examined using multivariate analysis of covariance with age, sex, education, and ICV as covariates, and false discovery rate correction for multiple comparisons was applied to control type II errors.

### 4.6 Exploratory analyses

For all FC analyses in BD‐I patients, information on the dose of medication was added as a covariate in the regression model to remove potential confounding effects. Four types of drugs were used, namely, mood stabilizers, atypical antipsychotics, antidepressants, and benzodiazepine, and medication dose was treated as a categorical variable, with no current use, current usage of one type, two types, three types, and four types of the above drugs being assigned as 0, 1, 2, 3, 4, respectively. Lithium load was specifically proposed given its reported neuroprotective effects.^41^ To further explore whether the kinds of medication used may have an effect on the FC differences we observed, we performed a multivariate analysis of covariance with age, sex, education, and ICV as covariates among patients using different kinds of medication.

In addition, a partial correlation analysis (two‐tailed) was performed to investigate the association between age of onset, illness duration, and symptom severity in patients and rsFC data extracted for each brain region which showed a significant difference in connectivity compared to HCs, with age, sex, and years of education as covariates. To further explore the relationship between FC and disease severity, we multivariate analysis of covariance with age, sex, education, and ICV as covariates among patients in different episode states (depression, mania, euthymia, and mixed episode) and with or without psychotic symptoms.

## 5 CONCLUSION

In conclusion, using a new seed‐based approach, significant differences have been observed in the FC patterns between subregions of the thalamus, prefrontal cortex, and DMN in patients with BD‐I, relative to HCs. These findings contribute to the growing body of evidence implicating the thalamus, particularly its anterior‐posterior subregions, in the underlying neuropathology of BD. Future studies to investigate the FC of subcortical subregions in different subtypes of BD and different mood states of BD will further add to understanding the pathophysiology of BD.

## AUTHOR CONTRIBUTIONS

Xipeng Long and Xiuli Wang conceptualized and designed the study, as well as provided continuous review and oversight of the running of the study along with Di Kong Xipeng Long, Xiuli Wang, Di Kong, Yuan Cao, Baolin Wu, Hongsheng Xie, Q.G., Neil Roberts, and Ziru Zhao recruited participants, collected, and interpreted data. Neil Roberts, Qiyong Gong, and Zhiyun Jia revised the manuscript. All authors have read and approved the final manuscript. The corresponding author had final responsibility for the decision to submit for publication.

## CONFLICT OF INTEREST STATEMENT

The authors declare no conflict of interest.

## ETHICS STATEMENT

All procedures involving human patients were approved by the Research Ethics Committees of West China Hospital of Sichuan University (study ethical approval number: 2020[331]). Written informed consent was obtained from all participants.

## Supporting information



Supporting Information

## Data Availability

The data that support the findings of this study are available from the corresponding author upon reasonable request.
