# Supplementary material for: Disorganized thalamic subregional functional connectivity in bipolar I disorder
Source: MedComm (2020). 2024 Oct 31;5(11):e771. doi: 10.1002/mco2.771 (PMC11527814; doi:10.1002/mco2.771)
Supplement: Supplementary file 1 — Supporting Information [file MCO2-5-e771-s001.doc]

**Supplementary Materials**

**Supplementary Methods**

1. **MRI data acquisition and preprocessing**
2. **Surface-based morphometry analysis**

**Supplementary Table 1**

**Supplementary Table 2**

**Supplementary Table 3**

**Methods**

**MRI data acquisition and preprocessing**

Magnetic Resonance Imaging (MRI) was performed on a 3.0 T system (Trio, Siemens Healthineers, Erlangen, Germany) equipped with a 32-channel head coil. Firstly, T1 weighted MR images were acquired by using a 3D MPRAGE sequence (TR = 2400 ms, TE = 2.01 s, TI = 1000 ms, flip angle = 8°, FOV = 224 by 224 mm, voxel size = 0.7 mm isotropic). Next, T2-weighted images were acquired using sampling perfection with application-optimized contrasts using different flip angle evolution sequences (TR = 3200 ms, TE = 565 ms, flip angle = 120°, FOV = 256 by 256 mm, slice thickness = 0.8 mm, and voxel size = 0.8 mm isotropic). These images were used to assist in making accurate pial surfaces, which excluded dura and blood vessels that were isointense to gray matter (GM) in the T1-weighted image. Finally, rs-fMRI data were collected using gradient-echo EPI sequence (TR =700 ms, TE = 37.8 ms, flip angle = 52°, FOV = 210 by 176 mm, slices = 64, thickness = 2.1 mm, voxel size = 2.1 mm isotropic).

The rs-fMRI data were collected in two parts, each lasting 5 minutes and including 415 volumes. As part of each acquisition, spin echo phase reversed images were acquired for registration with T1 images, and spin echo field maps were acquired to allow correction of bias fields. During all scans, participants were instructed to relax, keep their eyes closed and stay awake.

Image analysis was performed by using a Linux Ubuntu (18) computer on which are installed the HCPpipelines (v4.2.1), MATLAB MCR (v2017b) and R (v4) software. The minimal pipeline recommended by the Human Connectome Project for (HCP)-style data was used for data pre-processing. In brief, pre-processing of the structural data included gradient distortion correction, co-registration between the T1w and T2w images, bias field correction, which makes use of the inverse relationship between the signal intensities of the T1w and T2w images, and finally, registration of the subject’s native structural image to MNI space. Pre-processing of the functional data was performed by using the fMRIVolume and fMRISurface functional pipelines. FMRIVolume removes spatial distortions, realigns volumes to compensate for subject head motion, co-registers the fMRI data to the structural data, reduces the bias field, normalizes the 4D image to a global mean, and masks the data with a brain mask derived from the structural data. FMRISurface takes a volume time series and maps it to the standard CIFTI grayordinates space (see Glasser et al. [1] for more details). Next, the ICA-FIX pipeline was applied to remove artifacts. This pipeline includes a classifier that had been trained on the HCP young adult sample as distributed with FIX (FMRIB's ICA-based Xnoiseifier) and manual inspection of the data acquired in the present study indicated that no component had to be relabeled. Finally, the images were smoothed with a 6 mm full width at half maximum (FWHM) Gaussian kernel and a bandpass filter with 0.01-0.1 Hz was applied.

**Surface-based morphometry analysis**

To explore whether the functional aberrations were accompanied by or associated with alterations in anatomy, the GM volume of the thalamus and its subregions were calculated by using Freesurfer software (V. 6.0). Thalamic subfield segmentation was performed using a module in FreeSurfer software that employs a tetrahedral mesh‐based probabilistic atlas built with histological data. By this algorithm, the volume of the twenty-five subfields were obtained, including anteroventral, laterodorsal, lateral posterior, ventral anterior, ventral anterior magnocellular, ventral lateral anterior, ventral lateral posterior, ventral posterolateral, ventromedial, central medial, central lateral, paracentral, centromedian, parafascicular, paratenial, reuniens, mediodorsal medial magnocellular, medial lateral parvocellular, lateral geniculate, medial geniculate, limitans, pulvinar anterior, pulvinar medial, pulvinar lateral, pulvinar inferior, and reticular. All segmentation was visually verified following a quality control protocol that is similar to the ENIGMA protocol (http://enigma.ini.usc.edu/). In brief, segmentation of each subject was visually checked by two coauthors independently (XPL and XLW) and segmentation results judged to be incorrect were excluded (no subject). Multivariate analysis of covariance (MANCOVA) was used to test for thalamic subregion volume differences between groups with subregions entered as dependent variables, and the false discovery rate (FDR) correction used to correct for multiple testing. For subregions that showed significance after FDR correction, post hoc tests (using LSD) were employed to determine where the difference between groups was. Partial Eta Squared (η2) was calculated to estimate effect sizes.

**Reference:**

1. Glasser, M.F., et al.*, The minimal preprocessing pipelines for the Human Connectome Projec*t. Neuroimage, 2013**.** 80: p. 105-24.

**Supplementary Table 1. ANOVA tests comparing BD-I patients with different episode states.**

|  |  | FC, mean ± SE |  |  |  |
| --- | --- | --- | --- | --- | --- |
| Brain region | Episode state | BD-I (n=73) | F |  | P value (unadjusted) |
| Right DP and LSFG | Depression | 0.180 ± 0.032 | 6.460 | 0.227 | 0.001 |
|  | Mania | 0.419 ± 0.055 |  |  |  |
|  | Euthymia | 0.121 ± 0.052 |  |  |  |
|  | Fixed | 0.340 ± 0.091 |  |  |  |
| Left DP and LSFG | Depression | 0.203 ± 0.031 | 6.944 | 0.240 | <0.001 |
|  | Mania | 0.420 ± 0.052 |  |  |  |
|  | Euthymia | 0.118 ± 0.019 |  |  |  |
|  | Fixed | 0.357 ± 0.184 |  |  |  |
| Right DA and LAng | Depression | 0.198 ± 0.028 | 6.499 | 0.228 | 0.001 |
|  | Mania | 0.407 ±0.047 |  |  |  |
|  | Euthymia | 0.146 ± 0.044 |  |  |  |
|  | Fixed | 0.296 ± 0.078 |  |  |  |
| Left DA and LAng | Depression | 0.256 ± 0.026 | 5.121 | 0.189 | 0.003 |
|  | Mania | 0.424 ± 0.045 |  |  |  |
|  | Euthymia | 0.193 ± 0.042 |  |  |  |
|  | Fixed | 0.303 ± 0.074 |  |  |  |
| Right DP and LMCC | Depression | 0.246 ± 0.031 | 6.998 | 0.241 | <0.001 |
|  | Mania | 0.511 ± 0.054 |  |  |  |
|  | Euthymia | 0.238 ± 0.051 |  |  |  |
|  | Fixed | 0.408 ± 0.089 |  |  |  |
| Left DP and LMCC | Depression | 0.234 ± 0.029 | 8.455 | 0.278 | <0.001 |
|  | Mania | 0.502 ± 0.050 |  |  |  |
|  | Euthymia | 0.224 ± 0.047 |  |  |  |
|  | Fixed | 0.415 ± 0.083 |  |  |  |
| Right DA and RPre | Depression | 0.434 ± 0.03 | 4.535 | 0.171 | 0.006 |
|  | Mania | 0.637 ± 0.052 |  |  |  |
|  | Euthymia | 0.407 ± 0.049 |  |  |  |
|  | Fixed | 0.528 ± 0.086 |  |  |  |
| Left DA and RPre | Depression | 0.409 ± 0.029 | 5.182 | 0.191 | 0.003 |
|  | Mania | 0.609 ± 0.05 |  |  |  |
|  | Euthymia | 0.383 ± 0.047 |  |  |  |
|  | Fixed | 0.559 ± 0.083 |  |  |  |

BD-I, bipolar disorder type I; SE, standard error; LSFG, left superior frontal gyrus, DP, dorsal posterior; DA, dorsal anterior; LAng, left angular gyrus; LMCC, left middle cingulate cortex; RPre, right precuneus.

**Supplementary Table 2. ANOVA tests comparing BD-I patients with different medication states.**

|  |  | FC, mean ± SE |  |  |  |
| --- | --- | --- | --- | --- | --- |
| Brain region | Medication state | BD-I (n=73) | F |  | P value (unadjusted) |
| Right DA and LAng | 0 | 0.359 ± 0.065 | 4.680 | 0.268 | 0.001 |
|  | 1 | 0.397 ±0.065 |  |  |  |
|  | 2 | 0.172 ± 0.035 |  |  |  |
|  | 3 | 0.161 ± 0.034 |  |  |  |
|  | 4 | 0.381 ± 0.065 |  |  |  |
| Right VA and RPre | 0 | 0.469 ± 0.074 | 3.630 | 0.221 | 0.006 |
|  | 1 | 0.659 ± 0.075 |  |  |  |
|  | 2 | 0.328 ± 0.040 |  |  |  |
|  | 3 | 0.371 ± 0.039 |  |  |  |
|  | 4 | 0.519 ± 0.074 |  |  |  |
| Left VA and RPre | 0 | 0.460 ± 0.072 | 4.468 | 0.259 | 0.001 |
|  | 1 | 0.619 ± 0.072 |  |  |  |
|  | 2 | 0.285 ± 0.039 |  |  |  |
|  | 3 | 0.335 ± 0.037 |  |  |  |
|  | 4 | 0.479 ± 0.072 |  |  |  |

BD-I, bipolar disorder type I; SE, standard error; DA, dorsal anterior; VA, ventral anterior; LAng, left angular gyrus; RPre, right precuneus.

**Supplementary Table 3. Simple effect tests comparing rsFC between each thalamic subregions in each group.**

| **Regions** | **Group** | **DP**  **P Value** | **VP**  **P Value** | **VA**  **P Value** | **DA**  **P Value** |
| --- | --- | --- | --- | --- | --- |
| LACC | BD I | < VA *p*< 0.001  < DA *p*< 0.001 | < VA *p*< 0.001  < DA *p*< 0.001 | < DA *p*< 0.001 |  |
|  | HCs |  | < DP *p*= 0.012  < VA *p*= 0.010  < DA *p*= 0.037 |  |  |
| LSFG | BD I |  | < DP *p*= 0.012  < DA *p*< 0.001 | < DA *p*= 0.035 |  |
|  | HCs |  | < DP *p*< 0.001 | < DP *p*= 0.045  < VA *p*< 0.001 |  |
| LMFG | BD I | < VA *p*= 0.017 | < VA *p*= 0.11  < DA *p*< 0.001 |  |  |
|  | HCs |  | < DP *p*< 0.001 | < DP *p*= 0.001 | < DP *p*= 0.002 |
| Lang | BD I |  | < DP *p*< 0.001  < VA *p*= 0.002  < DA *p*< 0.001 | < DA *p*= 0.017 |  |
|  | HCs |  | < DP *p*< 0.001 | < DP *p*< 0.001 | < DP *p*< 0.001 |
| LMCC | BD I | < VA *p*< 0.001  < DA *p*< 0.001 | < DP *p*< 0.001  < VA *p*< 0.001  < DA *p*< 0.001 |  |  |
|  | HCs |  | < DP *p*< 0.001  < VA *p*= 0.014 | < DA *p*= 0.005 |  |
| RPre | BD I |  | < DP *p*< 0.001  < DA *p*< 0.001 | < DA *p*< 0.001 |  |
|  | HCs |  | < DP *p*< 0.001 | < DP *p*< 0.001  < DA *p*< 0.001 | < DP *p*< 0.001 |

BD I, bipolar disorder type I; HCs, healthy controls; rsFC, resting-state functional connectivity; MNI, Montreal Neurological Institute; L, left; R, right; LACC. Left anterior cingulate cortex; RSFG, right superior frontal gyrus; RPre, right precuneus; LSFG, left superior frontal gyrus; LAng, left angular gyrus; LMCC, left middle cingulate cortex; DP, dorsal posterior; VP, ventral posterior; VA, ventral anterior; DA, dorsal anterior.
